# Muscle Mass, Muscle Morphology and Bone Health Among Community-Dwelling Older Men: Findings from the Hertfordshire Sarcopenia Study (HSS)

**DOI:** 10.1007/s00223-018-0388-2

**Published:** 2018-01-25

**Authors:** H. P. Patel, A. Dawson, L. D. Westbury, G. Hasnaoui, H. E. Syddall, S. Shaw, A. A. Sayer, C. Cooper, E. M. Dennison

**Affiliations:** 1 MRC Lifecourse Epidemiology Unit, University Hospital Southampton, University of Southampton, Tremona Road, Mail point 95, Southampton, SO16 6YD UK; 2 Academic Geriatric Medicine, University of Southampton, Tremona Road, Southampton, SO16 6YD UK; 3 National Institute for Health Research Southampton Biomedical Research Centre, University of Southampton and University Hospital Southampton NHS Foundation Trust, Southampton, SO16 6YD UK; 4 AGE Research Group, Institute of Neuroscience, Newcastle, UK; 5 NIHR Newcastle Biomedical Research Centre, Newcastle upon-Tyne NHS Foundation Trust and Newcastle University, Newcastle, UK; 6 National Institute for Health Research Musculoskeletal Biomedical Research Unit, University of Oxford, Oxford, UK

**Keywords:** Bone–muscle relationship, Lean mass, Muscle morphology, Osteoporosis

## Abstract

Sarcopenia and osteoporosis are associated with poor health outcomes in older people. Relationships between muscle and bone have typically been reported at a functional or macroscopic level. The aims of this study were to describe the relationships between muscle morphology and bone health among participants of the Hertfordshire Sarcopenia Study (HSS). 105 older men, mean age 72.5 (SD 2.5) years, were recruited into the HSS. Whole body lean mass as well as appendicular lean mass, lumbar spine and femoral neck bone mineral content (BMC) and bone mineral density (BMD) were obtained through dual-energy X-ray absorptiometry scanning. Percutaneous biopsy of the *vastus lateralis* was performed successfully in 99 participants. Image analysis was used to determine the muscle morphology variables of slow-twitch (type I) and fast-twitch (type II) myofibre area, myofibre density, capillary and satellite cell (SC) density. There were strong relationships between whole and appendicular lean body mass in relation to femoral neck BMC and BMD (*r* ≥ 0.43, *p* < 0.001). Type II fibre area was associated with both femoral neck BMC (*r* = 0.27, *p* = 0.01) and BMD (*r* = 0.26, *p* = 0.01) with relationships robust to adjustment for age and height. In unadjusted analysis, SC density was associated with whole body area (*r* = 0.30, *p* = 0.011) and both BMC (*r* = 0.26, *p* = 0.031) and area (*r* = 0.29, *p* = 0.017) of the femoral neck. We have demonstrated associations between BMC and changes in muscle at a cellular level predominantly involving type II myofibres. Interventions targeted at improving muscle mass, function and quality may improve overall musculoskeletal health. Larger studies that include women are needed to explore these relationships further.

## Introduction

Sarcopenia is associated with disability, impaired quality of life and mortality in older people [1]. Health sequelae associated with sarcopenia include physical frailty, type II diabetes, obesity and osteoporosis [2–4]. The combination of mobility disability and osteoporosis increases the risk of falls and subsequent fracture that are also independently associated with significant morbidity, chronic disability, need for long-term care, high health care costs and mortality [5, 6]. Several pathophysiological mechanisms contribute to the development of sarcopenia including muscle denervation, mitochondrial dysfunction, declines in neurohormonal drive, inflammation, impaired satellite cell (SC) function and/or number, physical inactivity and undernutrition [4, 7, 8]. The changes contribute to an unfavourable decline in muscle mass, quality and consequently muscle function. Muscle mass is determined by myofibre size and number. At a cellular level, sarcopenia appears to be associated with a global loss of both type I, slow-twitch and type II, fast-twitch myofibres with a preferential loss and atrophy of type II fibres [9–11].

Osteoporosis is common in both genders and with increasing age. It is characterised by abnormalities of both trabecular and cortical bone, demonstrated on DXA scan as lower bone mineral density (BMD) as well as content (BMC). This leads to a progressive decline in the ability of bone to resist deformation from low energy trauma [5]. Bone and skeletal muscle share the same mesenchymal origin and respond to similar trophic cues from hormones, growth factors and inflammatory mediators [12, 13]. For example, bone mass is influenced by mechanical loading produced not only by gravity but by body mass and skeletal muscle contraction. As a consequence, dysfunction of one tissue can affect the other and changes in bone mass will follow declines in body weight, muscular mass and strength [14, 15]. Several studies have examined the association between low skeletal muscle mass, sarcopenia and osteoporosis [3, 14, 16, 17]. For example, in a study of women who had sustained a hip fracture, DiMonaco et al. showed that a low appendicular lean mass index (aLMi: aLM/height^2^) was significantly associated with osteoporosis [18]. In a further study of both men and women, the same authors found a high prevalence of low aLMi, previously referred to as sarcopenia, post hip fracture [19]. Analysis of approximately 2000 community-dwelling older men by Yu et al. showed that a diagnosis of sarcopenia (using the Asian Working Group on Sarcopenia in Older People [AWGS] diagnostic algorithm) was independently associated with increased fracture risk over a 12-year follow-up and this risk substantially increased with a simultaneous diagnosis of osteoporosis [20]. Men are known to have higher morbidity and mortality rates from osteoporotic-related fracture associated, in part, with low treatment rates and a higher likelihood of not receiving treatment [21].

Few studies have explored cellular changes in muscle and their association with measures of bone health such as BMD or BMC. One study in a murine model of senile osteoporosis showed that cross-sectional areas of both type I and type II fibres were lower in soleus muscle [22]. In a study of older women aged 60–85 with osteoporosis undergoing hip arthroplasty, morphology analysis revealed preferential type II fibre atrophy that was highly correlated with BMD [23]. To our knowledge, there are no population-based studies of muscle morphology and bone health in older men. Our aims for this study were to explore the relationship between muscle morphology and bone health as assessed by dual-energy X-ray absorptiometry (DXA) among healthy community-dwelling older men aged 68–77 years participating in the Hertfordshire Sarcopenia Study (HSS).

## Methods

### Study Participants

The Hertfordshire Sarcopenia Study (HSS) was designed to investigate life course influences on muscle morphology, mass and strength in community-dwelling older people. We recruited 105 healthy community-dwelling older men aged between 68 and 77 years who previously participated in the Hertfordshire Cohort Study (HCS) [24]. Participants were characterised in terms of their physical performance, body composition, muscle morphological parameters and sarcopenia status. Inclusion and exclusion criteria and study methods have been previously described in detail [25]. The Hertfordshire Research Ethics Committee approved the study (approval number 07/Q0204/68) and each participant gave written informed consent. Investigations on participants were conducted in accordance with the principles expressed in the Declaration of Helsinki.

### Anthropometry, Bone Health, Body Composition and Physical Performance Measurements

Height in centimetres (cm) and weight in kilograms (kg) were measured once. Body composition was assessed by dual-energy X-ray absorptiometry (DXA) (Hologic Discovery, software version 12.5) and was performed on all participants to quantify area, BMC and BMD relating to the whole body, total lumbar spine and total femoral neck, as well as total lean mass (kg) and appendicular lean mass (aLM) (kg). Isometric grip strength (kg) was measured three times in each hand using a Jamar handheld hydraulic dynamometer (Promedics, UK) and the highest reading of six was used for analysis [26]. A validated battery of tests was used to assess lower limb muscular function that included: five timed chair rises, a 6-m timed up-and-go test and customary walking speed over 3 ms [27–29]. Both the DXA machine and Jamar dynamometer were calibrated at regular intervals throughout the study.

### Muscle Biopsy

Percutaneous muscle biopsies of the *vastus lateralis* were conducted after an overnight fast under local anaesthetic using a Weil-Blakesley conchotome [30]. Of the 105 men, 102 were eligible for the procedure; three were ineligible as they were taking medication that might influence subsequent wound healing (*n* = 2) or predispose to haematoma formation (*n* = 1) and biopsies from a further three participants were not suitable for analysis [31]. Therefore, 99 had muscle biopsies that successfully yielded sufficient tissue for morphological analysis.

### Immunohistochemistry

**Fig. 1:**
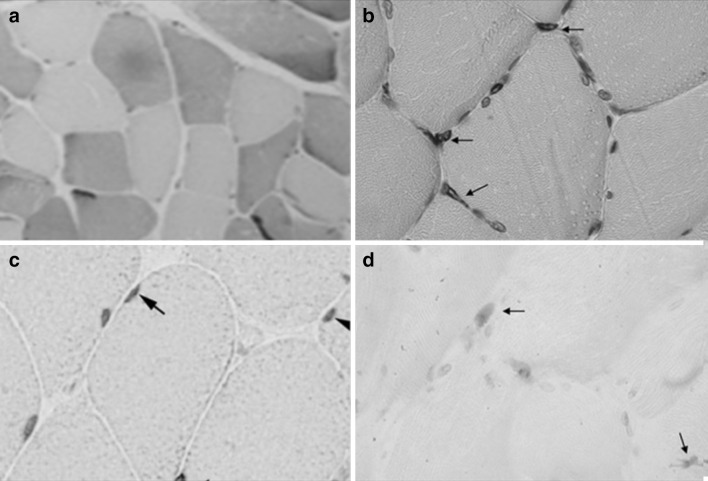
Muscle morphology. **a** Serial cross section showing differential fibre staining. Dark-stained fibres represent type II, fast-twitch fibres stained with anti-myosin-fast antibody (clone MY32, 1:6000 Sigma-Aldrich). **b** Serial cross section showing capillary staining. Capillaries are stained brown and arrowed at the peripheries of the myocyte (Ulex Europaeus Agglutinin 1, 1:200, Vector laboratories, UK), are arrowed and are located at the periphery of the myocyte. **c** Hematoxylin staining showing normal myonuclei. **d** Serial cross section showing satellite cells (SC). SC have been stained red at the peripheries of the myocyte, are arrowed and are clearly differentiated from myonuclei

The protocol and primary antibodies used for histochemical analysis on muscle tissue have been previously described [31]. The following morphological parameters were obtained: type I and II myofibre density (fibres/mm^2^) and myofibre cross-sectional areas (CSA) (µm^2^) (Fig. 1a). Fast fibre proportions were expressed as a percentage of total fibres. The total number of muscle fibres and capillaries was quantified from a given tissue area. Within this area, capillary density (capillaries per mm^2^) and capillary: fibre ratio were derived (Fig. 1b). Satellite cells (SCs) were identified in separate tissue sections that were counterstained with haematoxylin to differentiate myonuclei (Fig. 1c, d). SCs were quantified as follows: SC density [cells/mm^2^] and SC to fibre ratio. Muscle morphology parameters were analysed in all samples by an observer who was blinded to the participants’ anthropometry, body composition and sarcopenia status.

### Statistical Analysis

Normality was checked through visual inspection of histograms. Skewed distributions were log-transformed or square-root transformed (if included zero). Body mass index (BMI) was calculated by dividing weight (kg) by the square of height (m).

Participant characteristics were described using summary statistics. The associations between muscle morphology, lean mass parameters and bone outcomes were examined using Pearson correlations. Partial correlations (adjusting for age and height) were examined for relationships with significant (*p* ≤ 0.05) Pearson correlations. The analysis sample consisted of the 99 participants who underwent muscle biopsy. All analyses were conducted using Stata release 14 (STATA Corp, College Station, Texas, USA).

## Results

### Participant Characteristics

Participant characteristics as well as statistics for the bone parameters and fibre morphology of the 99 HSS men are presented in Table 1. Mean (SD) age was 72.4 (2.4) years. The participants had mean weight, BMI, total lean mass and aLM values of 82.7 (12.6) kg 27.2 (3.6) kg/m^2^, 56.4 (6.5) kg and 24.1 (3.1) kg, respectively.

**Table 1:** Participant characteristics

	Mean (SD)
Age (years)	72.4 (2.4)
Height (cm)	174.1 (6.5)
Weight (kg)	82.7 (12.6)
BMI (kg/m^2^)	27.2 (3.6)
Lean mass (kg)	56.4 (6.5)
Appendicular lean mass (kg)	24.1 (3.1)
Bone measurements	
Whole body total area (cm^2^)	2252.0 (164.1)
Whole body total BMC (g)	2806.1 (434.0)
Whole body total BMD (g/cm^2^)	1.24 (0.14)
Total lumbar spine area (cm^2^)	70.1 (5.8)
Total lumbar spine BMC (g)	77.0 (16.7)
Total lumbar spine BMD (g/cm^2^)	1.09 (0.19)
Total femoral neck area (cm^2^)	46.9 (4.2)
Total femoral neck BMC (g)	48.0 (7.8)
Total femoral neck BMD (g/cm^2^)	1.02 (0.14)
Myofibre morphology	
Number of Type I fibres counted	199.0 (95.5)
Type I fibre area (µm^2^)	4774.8 (1201.7)
Type I fibre density (fibres/µm^2^)	78.3 (28.0)
Number of Type II fibres counted	199.0 (95.5)
Type II fibre area (µm^2^)	3953.5 (1144.4)
Type II fibre density (fibres/µm^2^)	101.9 (38.3)
Type II fibre percentage	56.1 (13.1)
Satellite cell density (cells/mm^2^)^a,b^	3.7 (2.3, 6.4)
Satellite cells per fibre^a,b^	0.04 (0.02, 0.07)
Capillary density (capillaries/mm^2^)	146.5 (43.2)
Capillaries per fibre	1.3 (0.3)

^a^Median (lower quartile, upper quartile)

^b^30 missing values as 30 slides were unsuitable due to suboptimal SC staining

### Relationships Between Lean Mass Measures and Bone Outcomes

**Fig. 2:**
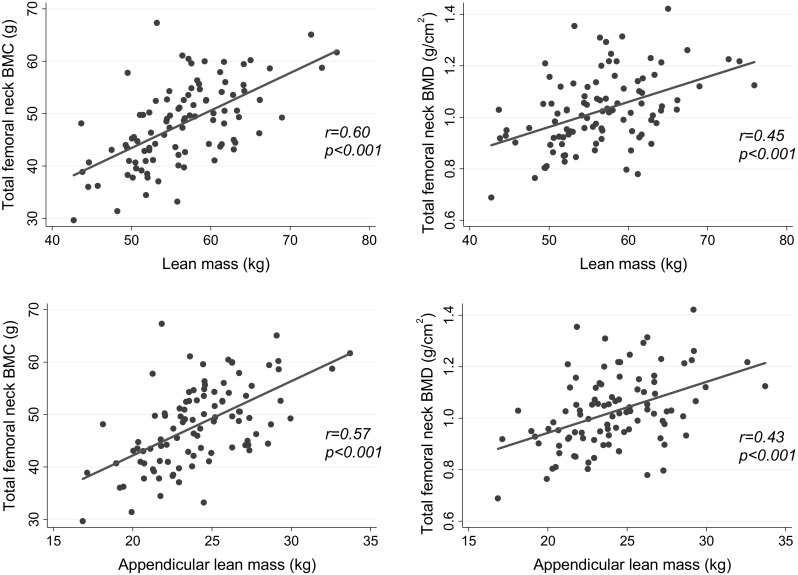
Unadjusted associations between total lean mass and appendicular lean mass, in relation to total femoral neck BMC and BMD. All associations were robust to adjustment for age and height. r: Pearson correlation coefficient, p: *p* value

Total lean and aLM were strongly associated (*p* < 0.01) with area, BMC and BMD at the whole body, lumbar spine and femoral neck in unadjusted analyses (Table 2). Unadjusted associations between lean mass and aLM, in relation to femoral neck BMC and BMD are presented in Fig. 2. After adjustment for age and height, aLM was not associated with lumbar spine area or BMC, or femoral neck area; lean mass was not associated with lumbar spine area; but the other associations remained significant (*p* < 0.05) (Table 3).

**Table 2:** Pearson correlations between muscle morphology, lean mass and bone parameters

Muscle parameter	Whole body			Total lumbar spine			Total femoral neck		
	Area	BMC	BMD	Area	BMC	BMD	Area	BMC	BMD
Slow fibre area	0.14	0.09	0.03	0.18	0.09	0.08	0.15	0.21	0.13
*p* value	0.167	0.378	0.746	0.113	0.441	0.427	0.137	0.045	0.214
Slow fibre count	− 0.15	− 0.07	− 0.03	0.00	0.10	0.09	− 0.03	− 0.14	− 0.15
*p* value	0.137	0.477	0.785	0.998	0.365	0.381	0.758	0.181	0.128
Slow fibre density	− 0.07	− 0.10	− 0.10	− 0.07	0.00	0.02	0.00	− 0.14	− 0.16
*p* value	0.479	0.348	0.305	0.508	0.995	0.811	0.979	0.160	0.113
Fast fibre area	0.09	0.16	0.16	− 0.11	0.01	0.12	0.06	0.27	0.26
*p* value	0.410	0.134	0.129	0.331	0.942	0.264	0.546	0.010	0.010
Fast fibre count	− 0.08	0.02	0.09	0.04	0.10	0.03	− 0.07	− 0.06	− 0.04
*p* value	0.434	0.848	0.363	0.689	0.358	0.742	0.505	0.549	0.707
Fast fibre density	− 0.05	− 0.01	0.04	− 0.09	− 0.09	− 0.11	− 0.13	− 0.18	− 0.12
*p* value	0.623	0.895	0.698	0.395	0.399	0.277	0.192	0.072	0.222
Fast fibre percentage	0.04	0.05	0.07	− 0.01	− 0.05	− 0.08	− 0.08	0.00	0.05
*p* value	0.696	0.609	0.463	0.905	0.636	0.411	0.445	0.995	0.628
SC density^a^	0.30	0.21	0.08	0.12	0.06	0.10	0.29	0.26	0.15
*p* value	0.011	0.083	0.498	0.368	0.679	0.431	0.017	0.031	0.221
SC per fibre^a^	0.34	0.20	0.05	0.15	0.12	0.08	0.33	0.22	0.07
*p* value	0.004	0.091	0.670	0.273	0.367	0.501	0.006	0.072	0.598
Capillary density	− 0.05	− 0.07	− 0.08	0.09	0.07	0.02	0.04	− 0.11	− 0.15
*p* value	0.654	0.516	0.449	0.410	0.548	0.819	0.699	0.274	0.158
Capillaries per fibre	− 0.08	− 0.07	− 0.06	− 0.02	− 0.06	− 0.06	− 0.07	− 0.09	− 0.06
*p* value	0.464	0.523	0.555	0.890	0.604	0.595	0.523	0.370	0.543
Lean mass	0.79	0.58	0.30	0.34	0.34	0.34	0.42	0.60	0.45
*p* value	< 0.001	< 0.001	0.003	0.001	0.001	0.001	< 0.001	< 0.001	< 0.001
App lean mass	0.73	0.55	0.29	0.28	0.29	0.32	0.39	0.57	0.43
*p* value	< 0.001	< 0.001	0.004	0.009	0.006	0.001	< 0.001	< 0.001	< 0.001

*SC* satellite cell, *App* appendicular

^a^Square-root transformed for normality

**Table 3:** Partial Pearson correlations (after accounting for age and height) between muscle morphology, lean mass and bone parameters

Muscle parameter	Whole body			Total lumbar spine			Total femoral neck		
	Area	BMC	BMD	Area	BMC	BMD	Area	BMC	BMD
Slow fibre area								0.12	
*p* value								0.246	
Fast fibre area								0.31	0.26
*p* value								0.003	0.011
Sat cell density^a^	0.13						0.14	0.16	
*p* value	0.307						0.255	0.199	
Sat cells per fibre^a^	0.08						0.14		
*p* value	0.522						0.279		
Lean mass	0.69	0.48	0.29	0.11	0.24	0.31	0.21	0.53	0.46
*p* value	< 0.001	< 0.001	0.004	0.331	0.030	0.002	0.042	< 0.001	< 0.001
App lean mass	0.61	0.44	0.28	0.03	0.18	0.29	0.17	0.49	0.45
*p* value	< 0.001	< 0.001	0.006	0.820	0.107	0.004	0.098	< 0.001	< 0.001

*Sat* satellite, *App* appendicular

^a^Square-root transformed for normality

### Relationships Between Muscle Morphology Parameters and Bone Outcomes

**Fig. 3:**
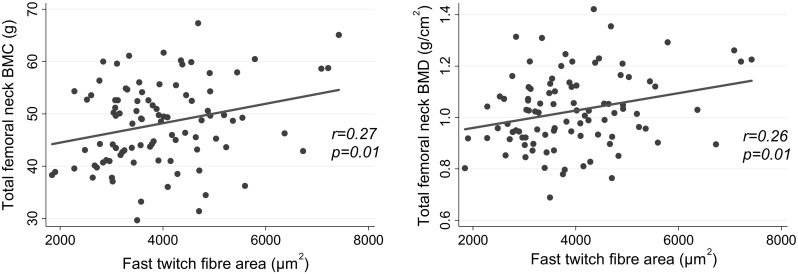
Associations between fast-twitch fibre area and femoral neck BMC and BMD. Associations were robust to adjustment for age and height. r: Pearson correlation coefficient, p: *p* value

**Fig. 4:**
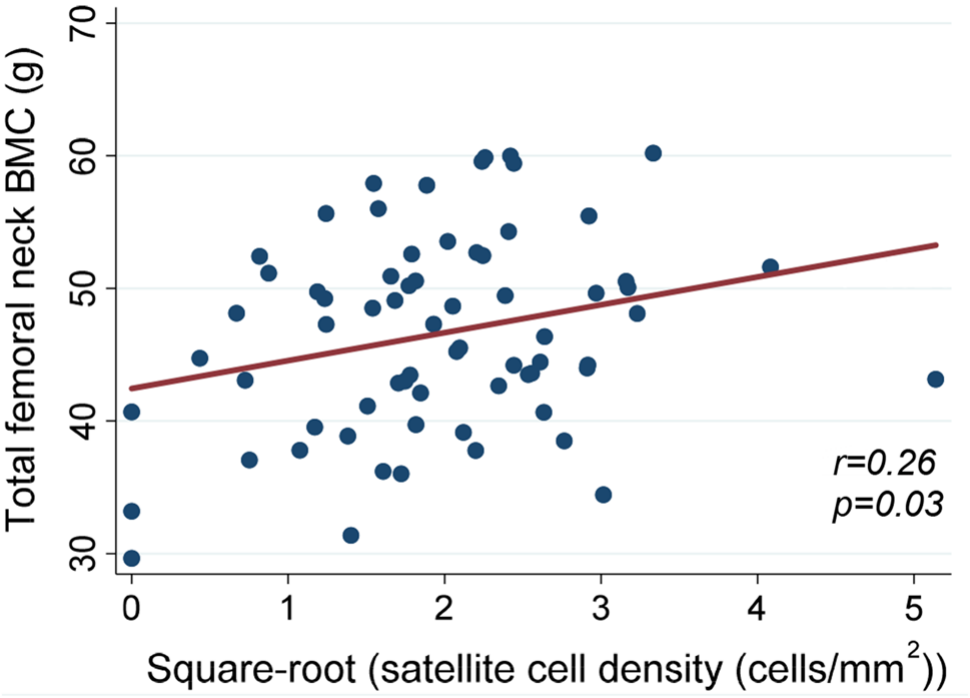
Association between total femoral neck BMC and the square root of satellite cell density. r: Pearson correlation coefficient, p: *p* value (Pearson correlation coefficient and *p* value derived after square-root transformation of satellite cell density to ensure normality)

Slow fibre area was associated with femoral neck BMC in the unadjusted analysis but this was not robust to adjustment for age and height (Tables 2, 3). Fast fibre area was associated with femoral neck BMC and BMD in the unadjusted analysis (Fig. 3) and after adjustment for age and height. In unadjusted analysis only, the number of SCs per fibre and SC density were both associated with whole body and femoral neck area; satellite cell density was also associated with femoral neck BMC (Fig. 4). All associations described above were positive (0 < *r* < 1).

## Discussion

In this unique study of a cohort of community-dwelling healthy older men, we identified significant relationships between whole body and appendicular lean mass and femoral neck BMC and BMD. Fast-twitch (type II) myofibre area was positively associated with femoral neck BMC and BMD. Satellite cell density and SC per fibre were positively associated with femoral neck BMC. The study of muscle tissue has immense benefits not only providing opportunity for molecular analysis, i.e. gene expression, but also allows the study of myofibre composition to assess the muscle size/strength relationship. This allows inferences to be made on how cell and molecular changes exert influence at the macroscopic/functional level.

Muscle and bone are and highly coupled from early embryonic development through adolescence, adulthood and involution in both structure and physical function [32]. Muscles and bones become larger and stronger from growth and undergo adaptive modelling not only in response to gravitational- and activity-based loading but also in response to nutrition, vitamins and essential nutrients such as vitamin D and calcium. These effects occur through mechanical and functional interactions mediated by bidirectional cross-talk between myokines and osteokines crucial to the bone–muscle secretome [33].

We have previously reported that muscle size is associated with bone size and strength measured by peripheral quantitative computerised tomography (pQCT) in women and men [34]. These findings support the mechanostat hypothesis which suggests that changes in bone mass with ageing generally follow the age-related change in muscular mass as well as strength [35, 36]. The results from our current study are in broad agreement with several previous reports describing the associations between lean mass and BMD. For example, in a study of older men and women aged 60–92 by Kirchengast and Huber, lean mass correlated significantly with whole body and femoral neck BMD in men. Furthermore, sarcopenic men (defined as those with low aLMI) were more likely to have osteopenia and osteoporosis than women [37]. Similarly, in another study of 679 older men aged 40–79, low aLM and aLMi was associated significantly with lower areal BMD. Again, men with sarcopenia (defined as low aLMi) were more likely to have osteoporosis [38]. In further support of our findings, a study of 198 men aged 60 and over by Pereira et al. concluded that low lean mass, low aLMi as well as sarcopenia (using the EWGSOP diagnostic algorithm) predicted lower BMD [39]. In a cross-sectional study of older men and women aged 73–93 years, Visser et al. showed that muscle mass was positively associated with total body, upper and lower limb BMD in men [40]. The relationships between lean mass and BMD at the lumbar spine and the hip have also been seen in older post-menopausal women [18, 40–43]. The clinical consequences of low lean mass on bone have also been explored previously. For example, men who sustained hip fracture were more likely to be sarcopenic as defined by low aLMi [19, 44].

Muscle mass is a function of fibre number and area. Myofibre cross-sectional areas are maintained by the myonuclear domain; the cytoplasmic area directly influenced by a myonucleus [45]. Since myofibres are syncytial, it follows that the viability and protein synthetic capability of the myonuclei will depend on a host of systemic, local and paracrine cues as well as renewal by satellite cells after myofibrillar damage. We speculate that an increase in type II, fast-twitch, myofibre area is consistent with an attainment and maintenance of greater lean mass. This is reflected in the positive associations we see with femoral neck BMC and BMD. However, we did not observe any associations between type I or type II fibre area and either whole body or lumbar spine BMC or BMD. The exact reasons for this are unclear. However, in support of our findings, a study of older women aged 65–85 undergoing hip arthroplasty showed that there was a preferential type II fibre atrophy in skeletal muscle obtained from the vastus lateralis as well as a significant negative correlation between increased proportion of type II fibre atrophy and BMD [23]. Furthermore, in a mouse model (SAMP 6 phenotype) of senile osteoporosis, soleus muscle type I and type II fibres exhibited significant atrophy [22]. Satellite cells are quiescent unipotent stem cells that have the ability to differentiate into myogenic cell lineage in response to muscle injury [46, 47]. We suggest that our findings of a positive correlation between SC density and femoral neck BMC reflect the positive correlation between Type II fibre area and femoral neck BMC.

To our knowledge we are the first to report the association between type II fibres, satellite cell density and BMD or BMC at the femoral neck in men. Our study has several limitations that need to be acknowledged. First, the sample size was modest which influences statistical power. Second, histological techniques used to quantify the morphology parameters were open to observer error although consistent and rigorous methods were applied throughout the study in order to limit this possibility. Finally, the histological parameters measured may not accurately reflect the morphological changes occurring in muscle. We suggest that longitudinal studies would be helpful to more fully characterise the morphological changes that occur over time in the muscle of people with sarcopenia, osteopenia and osteoporosis.

Our study has a number of strengths. First, we have shown that it is feasible to obtain tissue from community-dwelling older men in the context of an epidemiological birth cohort. The advantage of this is that morphological data can be combined with the extensive phenotypic data that have already been collected. Second, the histology methods employed were based on tested protocols and can be applied to future studies that also include women. Our study shows that quantification of several morphological variables is possible using these methods.

## Conclusions

We have shown that there are significant relationships between muscle mass and bone mineral density and content. At a cellular level these relationships appear to arise from morphological changes that predominantly affect type II myofibres. Although, future studies would need to include women, our results suggest that attaining and maintaining higher total lean as well as appendicular muscle mass may prevent a decline in BMD and progression to osteoporosis. Identifying people with coexistent of sarcopenia and osteoporosis may be clinically important. These people represent an especially vulnerable group who are at high risk of falls, fractures and further morbidity and are therefore in need of interventions that combine pharmacological therapy, nutrition and physical activity to maintain muscle as well as bone strength and function.
